# De Facto Versus de Jure Political Institutions in the Long-Run: A Multivariate Analysis, 1820–2000

**DOI:** 10.1007/s11205-015-1204-2

**Published:** 2016-02-17

**Authors:** Peter Foldvari

**Affiliations:** 1 International Institute of Social History, Cruquiusweg 31, 1019 AT Amsterdam, The Netherlands; 2 Utrecht University, Drift 6, 3512 BS Utrecht, The Netherlands

**Keywords:** Democratization, De facto and de jure institutions, Canonical correlation, Polity IV, Vanhanen’s Index of Democracy, N40, O17

## Abstract

In this paper we use the components of the PolityIV project’s polity2 and Vanhanen’s Index of Democracy indicators to analyse the relationship between de jure and de facto political institutions from 1820 until 2000 with a canonical correlation method corrected for the sample selection bias. We find considerable fluctuation in the relationship between the two measures. After a moderate positive correlation found during the first half of the nineteenth century, the two measures become statistically unrelated until the 1940s. The relationship becomes strong and positive only in the second half of the twentieth century. The relationship between de jure and de facto political institutions hence can be described as a U-curve, reminiscent to an inverse Kuznets-curve.

## Introduction

The distinction between de jure and de facto institutions is a crucial element in understanding how institutions explain observed cross-country differences in socio-economic outcomes as emphasized in Feld and Voigt (2003), Pande and Udry (2006) and Voigt (2013). In line with past literature, we define de jure institutions as those comprising of official, formal rules, while de jure institutions are the ones that are enforced and followed in practice. Accordingly, de jure institutions are observable as rules, while de facto institutions are reflected by practices and outcomes. The proper measurement of institutions and the distinction between policies and institutions has become the next focal point in institutional economics as pointed out by Voigt (2013) and Robinson (2013). The purpose of this paper is two-fold. First, we empirically examine if two historical democracy measures that dominate scholarly usage, the polity2 of the PolityIV project (Marshall et al., 2012) and Index of Democracy (ID) by Vanhanen (2000, 2003) can be used as proxies of de jure and de facto institutional measures respectively. Using these two measures, we then investigate whether the relationship between de jure and de facto political institutions changed over the 1820–2000 period.^1^ We find that after a period of moderate positive correlation between the two measures in the first half of the nineteenth century, they become disconnected until about the 1940s when the link begins recovering, but a strong positive statistical relationship is present only from the 1970s on.

The rest of the paper is organized as follows. Section 2 presents a brief conceptual explanation for the difference between de jure and de facto institution and the related theoretical literature. In Sect. 3.1 we show why the polity2 and the ID indicators can be used as proxies of de jure and de facto political institutions. This is followed by the discussion of methodological and measurement issues in Sect. 3.2. Section 3.3 presents the observed secular trends where we find that the discrepancy between the two types of political institutions follows a non-linear U-shaped pattern, reminiscent of an inverse Kuznets curve. Section 4 summarizes the main findings of the paper.

## Literature Review

North (1991, p. 97) defines institutions as “humanly devised constraints that structure political, economic and social interactions”, which is somewhat restrictive. Hodgson (2006, p. 2.), suggests a more flexible approach instead by stating that “we may define institutions as systems of established and prevalent social rules that structure social interactions”. The role of good institutions is well-established empirically in the literature including legal origin and religion (La Porta et al. 1999; Shirley 2008), the risk of expropriation and the government’s commitment to respect private contracts (Hall and Jones 1999), or more generally, the rule of law (Hansson 2009). Recently, the focus of research turned toward the distinction between de jure and de facto institutions and the related measurement issues. As Feld and Voigt (2003) illustrate in their seminal paper, observed cross-country differences in economic growth are better explained by de facto judicial independence than by de jure rules. Robinson (2013) explicitly calls for the distinction between de facto enforcement and de jure rules citing the observed contradictions between de jure rules and de facto practices of land redistribution in the Trobriand-islands. Additionally, the possible effects of other de jure institutions, such as property rights, on economic performance are conditional on the state of de facto judicial independences (Voigt and Gutman 2013).

First of all, we adopt a definition for political institutions, since there is no consensus in the literature on what is meant by political institutions. By political institutions we mean those formal or informal rules, conventions and norms that govern and constraint the operation of the government, the operation of political organizations and the distribution of political power. Hence, political institutions include the rules of the selection of political executives, the limitations on the government’s power (especially with respect to its citizens) and legislation. De jure political institutions may take various forms such as customs, laws, and constitutions. Yet, these laws are not necessarily enforced, and uncodified rules may be in operation in a society. This set of rules, written or unwritten, that are actually enforced in a society and in practice shape the behaviour of political agents is labelled as de facto institutions.^2^ This distinction becomes especially useful when countries with comparable written political rules experience very different social and economic outcomes. One obvious example is the political system of the Soviet Union under Stalin. The Soviet Constitution of 1936, even though assigned a leading role to the communist party, also declared some fundamental rights to its citizens, including freedom of religion and universal direct suffrage. Yet, these de jure guarantees did not prevent Stalin from keeping his absolute power over the state and to run one of the most oppressive police-states of human history. Obviously, de facto political institutions are more useful in explaining the history of the Soviet Union than the de jure ones.

As for the existence and possible effects of discrepancies between de facto and de jure political institutions, both political economy and institutional economics offers theoretical explanations that predict certain interrelations between de facto and de jure political institutions and also their relationship with other social, political or economic variables.

We begin with the political economic explanation. The government, as agent, plays a fundamental role in shaping and enforcing the fundamental rules of interactions, which takes the form of formal laws and practices. Acemoglu and Robinson (2012) make a distinction of inclusive and extractive political institutions. While the former should reflect and enforce the interest of the majority, the latter supports extractive economic institutions that channel resources from the society toward the elite. But the composition of the political elite is not stationary (Acemoglu and Robinson 2006). When another political group challenges the establishment, the ruling elite will face the choice of either adopting reforms (changing formal rules) or to resist them at the risk of political instability. The historical process of regime changes is not automatic, though, and there is an underlying non-linearity in it: Robinson and Acemoglu (2002) find that regime changes are more likely to occur at intermediate levels of income inequality. Thereby they link their political economic model to the empirically observed phenomenon of the Kuzents curve.^3^ Their framework of inclusive versus extractive economic and political institutions can be linked to the concept of de jure and de facto political institution via the instability introduced by regime-changes. In cases when the elite are forced to make concessions, one should observe relatively quick changes in the de jure (formal) institutions, but just slow or even insignificant changes in the de facto institutional framework.^4^

Another explanation for the discrepancies between the de jure and de facto political institutions is offered by the institutional economics literature. Boettke et al. (2008) distinguish foreign-introduced exogenous, indigenously introduced exogenous and indigenously introduced endogenous institutions. Exogenous institutions are constructed and forced from above, either by an indigenous group or by a foreign power (colonizer or an international organization). Endogenous institutions, on the other hand, are the result of some spontaneous process originating from within the same society. Boettke et al. claim that these different institution types exhibit different degree of stickiness or ability to resist changes. Endogenous institutions can efficiently resist external influences for a very long time, while the foreign-induced ones can be discarded quickly once no external pressure is present. Hence, we can expect that the difference between de jure and de facto political institutions has grown with the globalization starting in the last decades of the nineteenth century, when non-European countries became increasingly subject to the expectations and directives of Western powers either directly (via colonization) or indirectly (by conditioning aid on political or economic reforms). Historical examples include the failed attempts by colonial powers to introduce their legislation in Sub-Saharan Africa [Pande and Udry (2006); Blewet (1995)], or the successful democratization of Germany and Japan after World War 2.

## Empirical Analysis

In this section we focus on the empirical analysis of the components of the polity2 and the ID indicators. First, in Sect. 3.1 we provide empirical evidence for the central claim of this paper, namely that the components of polity2 primarily reflect changes in de jure political institution while the components of ID can better be used as proxies of the de facto political rules and practices. In Sect. 3.2 we apply the method of canonical correlation to estimate the strength of the relationship between the components of the two democracy variables. Finally, Sect. 3.3 provides a brief overview of the secular trends in the relationship between the components of ID and polity2.

### Measurement Issues: Proxies for de Jure and de Facto Political Institutions

In this paper, we claim that even though both the Index of Democracy (ID) and the polity2 attempt to capture the same dimensions of democracy, namely competition and participation in the terminology of Dahl (1972), the former is more successful in measuring actual outcomes (de facto political institutions) by directly using statistics on voter turnout and the composition of parliaments, while the five components of polity2 are in fact better correlated with the formal rules and practices (de jure political institutions).^5^ We wish to stress that we do not argue that the two measures were created with the purpose of measuring de facto or de jure political institutions. What we claim is that due to the different methodology, they ex-post prove to be better proxies of either the de facto or the de jure political institutions and that this difference may be useful to gain some insight in the secular trend of the convergence and divergence between these two aspects of political institutions.^6^

In this section we use three empirical ways to show that the Polity IV project and the Vanhanen’s Index of Democracy capture different aspects of political institutions.^7^ One is based on the observation of main historical trends, the second is on a test for Granger causality and the last one is based on the correlation with existing measures of de jure political institutions.

**Fig. 1:**
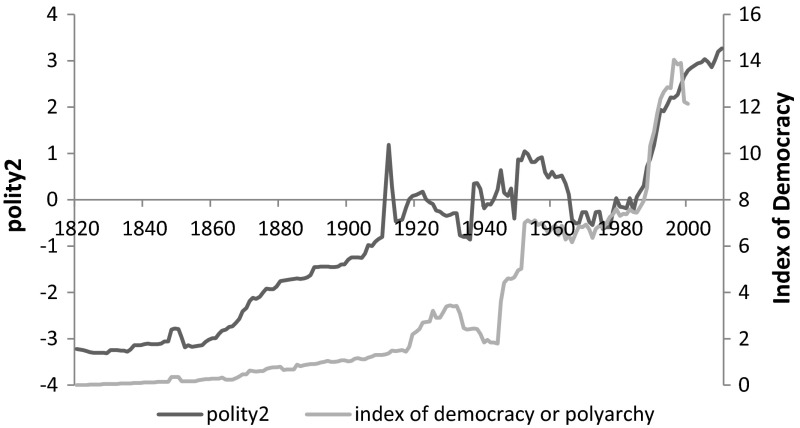
World average scores in different measures of the degree of democracy of political institutions, 1820–2010, Polity2 score of the Polity IV project (−10/+10) and the index of democracy (%). *Sources* the polity IV dataset by Marshall et al. (2012) and the polyarchy data by Vanhanen (2000, 2003)

Firstly, we look at the secular trends in democratization as suggested by the two measures (Fig. 1). The world averages of the polity2 aggregate suggests that the global democratization process already began in the mid-nineteenth century, while Vanhanen’s ID dates the start of the process at the mid-twentieth century. The two aggregate indicators seem to converge only after the 1950s. Consequently, it is questionable if we can speak of three global waves of democratization at all, a categorization suggested by Huntington (1991, 1993), since it is only confirmed by the polity2 but not by the ID. Huntington bases his classification of democracy based on changes in the de jure political institutions and considers the political elite’s behaviour as the main driving factor behind the third wave. This is clearly reflected by the polity2, but not by the ID.

**Fig. 2:**
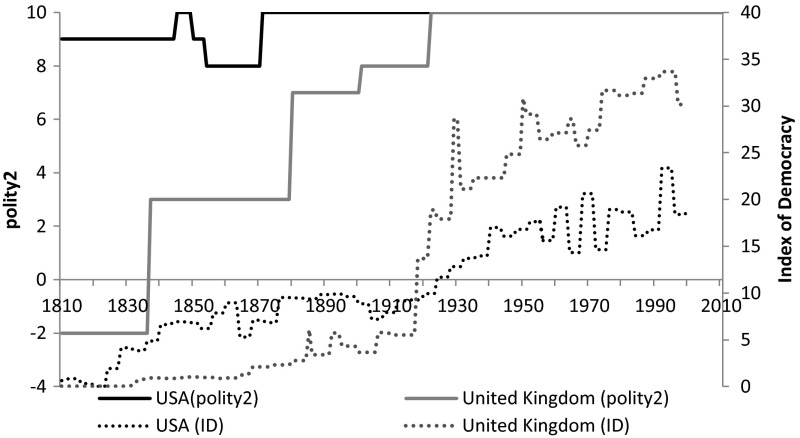
The polity2 and ID scores for the US and the UK, 1810–2010, Polity2 score of the Polity IV project (−10/+10) and the index of democracy (%). *Sources* the polity IV dataset by Marshall et al. (2012) and the polyarchy data by Vanhanen (2000, 2003)

The country-specific trends reveal even more peculiar differences in the underlying political institutional factors captured by the two indicators. Perhaps the most striking differences can be observed in the ranking of the USA versus Western European countries (the UK, the Netherlands, France and Switzerland) but since these are quite similar we focus on the USA–UK comparison now. Figure 2 reports the relative position of the United States and the United Kingdom in terms of democracy as reflected by the two measures. The Polity IV project assigns very high score to the USA during the first half of the nineteenth century, even though a considerable percentage of the population (Afro-Americans and the indigenous population) was still disfranchised. After the Civil War, the USA is constantly set at a maximum score of 10, which is only reached by the UK after World War 1. The Index of Democracy exhibits a fundamentally different picture: both countries have a clear trend of increasing democracy but the USA is overtaken by the UK around 1920 which coincides with the significant extension of political rights that resulted in more participation and competition. Actually almost all jumps in the UK series can be identified as an election. The 1837 case, for example, can be identified as the first election under Queen Victoria’s reign, the last case when the mandate of the Parliaments ended with the death of the monarch. Similarly the second positive change is in 1880, another general election, again with a liberal victory and the last positive change is the General Elections of 1923.

While one possible explanation for the observed trends could be that the polity2 is based too much on American concepts of democracy which results in USA serving as an etalon for the whole democratization process, we believe that the explanation lies elsewhere. The polity2 components are necessarily based on observable changes in the practices and laws as declared by the political elite. Even if the elite has genuine commitment toward democratization, polity2 will only reflect changes in de jure institutional framework, i.e. when there is a change in the written legal sources used for classification. This explains why we observe sudden jumps in the polity2 reflecting changes in laws, but it is very unlikely that de facto political institutions (or any other de facto institutions) could ever undergo changes so fast. Hence the moderate nature of changes in the Index of Democracy is already an indication that it does not measure the same aspect of democracy as polity2.

The second evidence is based on the analysis of causality, as defined by Granger (1969).^8^ Even if changes in the de jure institutions may not always be transplanted with success as Berlowitz et al. (2003) and Boettke et al. (2008) suggest, we can still expect some equilibrium between the de jure and de facto institutions (Aoki 2007). For such equilibrium to exists, however, it is necessary that at least one of the indicators adjusts. The possibly asymmetric behaviour of the two political indicators can be of use to indirectly test if our assumption is correct. We use the below dynamic panel specification to test for the direction of Granger-causality in two sub-samples: Sub-Saharan Africa and North-western Europe.

1 $$Y_{i,t} = \alpha + \sum\limits_{j = 1}^{5} {\beta_{j} Y_{i,t - j} } + \sum\limits_{j = 1}^{5} {\gamma_{k} X_{i,t - k} + } \;\eta_{i} + \lambda_{t} + u_{i,t}$$

where Y and X denote either the polity2 or the ID depending on the direction of causality being tested, *η* and *λ* denote the country and year specific unobserved effects and *u*_*i,t*_ is the random error term. The inclusion of year specific effects is important so that we exclude the possibility that a third, unobserved factor drives the relationship between polity2 and ID leading to spurious results. Since the ID can only measure changes in institutions when elections are allowed, for this test we only use those observations where ID > 0.

If polity2 indeed represents de jure and ID reflects de facto political institutions, then for regions where changes in the de jure political institutions are indigenous and are compatible with the local institutions (Berkowitz et al. 2003; Boettke et al. 2008), we should expect that changes in de jure institutions predate changes in the de facto political institutions. That is, if we are correct, changes in polity2 Granger-cause changes in ID. This is exactly what we obtain for North-western Europe (reported in the upper rows in Table 1): Table 1 suggests that in North-western Europe changes in the ID do not predict changes in the polity2 but they do vice versa.

**Table 1:** Granger causality test results from Eq. (1)

	Dependent variables	
	Index of democracy	Polity
*North-western Europe*		
Lags of index of democracy	0.000 (+)	0.596 (+)
Lags of polity2	0.000 (+)	0.000 (+)
*Sub-Saharan Africa*		
Lags of index of democracy	0.000 (+)	0.003^*^ (−)
Lags of polity2	0.360 (+)	0.000 (+)

Sample is limited to cases with ID > 0

If changes in de jure political institutions are mostly exogenous, however, just as the case is in Sub-Saharan Africa, we expect that changes in de jure institutions should not affect de facto institutions in any significant manner. What we found in the bottom rows of Table 1 is even more peculiar: while we find that changes in polity indeed do not Granger-cause changes in the ID, ID seem to have Granger-caused polity in the short-run only. The total, long-term effect of ID on polity is statistically not significant from zero in Sub-Saharan Africa. That is, changes in the polity2 in Sub-Saharan Africa only had a short-run, temporary effect on ID in Sub-Saharan Africa, but no long-run effect can be found at all.

The third evidence is direct. Fedderke et al. (2001) and Gwenhamo et al. (2012) estimated an index of de jure political institutions (political freedom) for South Africa and Zimbabwe respectively.^9^ They explicitly take only de jure institutional changes into account hence their estimates can be used for a cross check. If our claim is correct, then their political freedom (PF) index should be correlated more strongly with the polity aggregate than with the ID.

We report the correlation coefficients in Table 2. Because of the presence of a trend or a possible non-stationarity, we report correlation between first differences. In Table 2 we find that in South Africa changes in the polity2 measure were positively and statistically significantly (at 1 %) correlated with changes in de jure political freedom measure by Fedderke et al. (2001), while the correlation with the ID is significant only at 5 %. The pattern is somewhat different in Zimbabwe, where we find that, despite the low number of observations, the polity2 is positively correlated with the Political Freedom by Gwenhamo et al. (2012) at 5 %, and the correlation coefficients between ID and Political Freedom is not only statistically insignificant at 10 % but it even turns out to be negative.

**Table 2:** Linear correlation coefficients among different indicators of political institutions (a) (South Africa, 1936–97),* N* = 62, (b) (Zimbabwe, 1981–2000), * N* = 20

	ΔPF	ΔPolity2	ΔID
( *a*)			
ΔPF	1		
ΔPolity2	0.497 (0.000)	1	
ΔID	0.257 (0.044)	0.354 (0.005)	1
(*b*)			
ΔPF	1		
ΔPolity2	0.553 (0.011)	1	
ΔID	−0.259 (0.269)	−0.048 (0.841)	1

*p*-Statistics reported in parenthesis

Besides conceptual issues, one also needs to cope with some technical problems when measuring democracy in a multidimensional perspective. The first important issue is the level of measurement. Most institutional indicators are measured on nominal scale (like the components of the polity2 score), which can usually be converted to an ordinal scale based on some theoretical expectations as done by Treier and Jackman (2008, see Table 3).

**Table 3:** Components of the polity2 index and coding rules

Variables	Possible outcomes	Values	Weight in polity2	Implied order
XRCOMP Competitiveness of Executive Recruitment	Election	3	2	4
	Transitional	2	1	3
	Selection	1	−2	1
	Unregulated	0	0	2
XROPEN Openness of Executive Recruitment	Open (“Election”)	4	1	6
	Dual: hereditary and election	3	1	5
	Dual: hereditary and designation	2	−1	2
	Closed	1	−1	1
	Unregulated	0	0	4
	Open (“no election”)	4	0	3
XCONST Constraint on Chief Executive	Parity or subordination	7	4	7
	Intermediate 1	6	3	6
	Substantial limitation	5	2	5
	Intermediate 2	4	1	4
	Slight moderation	3	−1	3
	Intermediate 3	2	−2	2
	Unlimited authority	1	−3	1
PARCOMP Competitiveness of Political Participation	Competitive	5	3	6
	Transitional	4	2	5
	Factional	3	1	4
	Restricted	2	−1	2
	Suppressed	1	−2	1
	Not applicable	0	0	3
PARREG Regulation of participation	Regulated	5	0	3
	Multiple identity	2	0	3
	Sectarian	3	−1	2
	Restricted	4	−2	1
	Unregulated	1	0	3

*Source* Table 1 in Treier and Jackman (2008, p. 204), and Marshall et al. (2013)

The polity project addresses this problem by assigning arbitrary numbers (weights) to different outcomes and sum them up to an aggregate measure labelled as polity2 in Polity IV (Table 3).^10^ Numerous studies use this aggregate measure as an explanatory variable even though, unless the arbitrary weighting accidently coincides with the theoretically correct one, this practice leads to an omitted variable problem and biased coefficient estimates.

Another issue is the inclusion of redundant variables as a result of arbitrary aggregation methods. The polity2 score is the sum of the weighted components, which completely neglects the commonalities between the components reflected by their correlations (see Tables 4 and 5).

**Table 4:** Spearman rank correlation coefficients between components of the polity2 score in 2000

	XRCOMP	XROPEN	XCONST	PARREG	PARCOM
XRCOMP	1				
XROPEN	0.884	1			
XCONST	0.840	0.691	1		
PARREG	0.801	0.629	0.808	1	
PARCOM	0.790	0.691	0.839	0.784	1

N = 151, we adopted the same ranking as Table 1 in Treier and Jackman (2008)

**Table 5:** Spearman rank correlation coefficients between components of the polity2 score in 1900

	XRCOMP	XROPEN	XCONST	PARREG	PARCOM
XRCOMP	1				
XROPEN	0.853	1			
XCONST	0.533	0.498	1		
PARREG	0.560	0.463	0.513	1	
PARCOM	0.578	0.575	0.502	0.719	1

N = 51, we adopted the same ranking as Table 1 in Treier and Jackman (2008)

Adding up these components hence leads to a double counting resulting in an aggregate component that has more variance than it should have if it were correctly representing the underlying latent factor of democracy. The same applies to the multiplicative aggregation adopted by Vanhanen who creates his aggregate Index of Democracy by multiplying observed data on participation (voter turnout) and competition (one minus the share of the winning party in the parliament). The multiplicative aggregation assures that only countries with a balanced performance in both aspects will have a high ID score, but there is no further reason to prefer it above the additive aggregation.

### A canonical Correlation Analysis

In this section we briefly introduce the canonical correlation analysis with a correction for sample selection bias, which is used to correct for the changing number of observations in the sample. Canonical correlation analysis is designed to find those linear combinations of two groups of variables (the components of the polity2 and ID aggregates) that maximize the correlation among them. If the two groups are not related, the canonical correlation coefficient should approach zero. When the canonical correlation is different from zero, the individual weights are indicative of which of the component variables dominate the relationship. In this way, canonical correlation also deals with the possible redundancy, since redundant components will have zero weight.

**Fig. 3:**
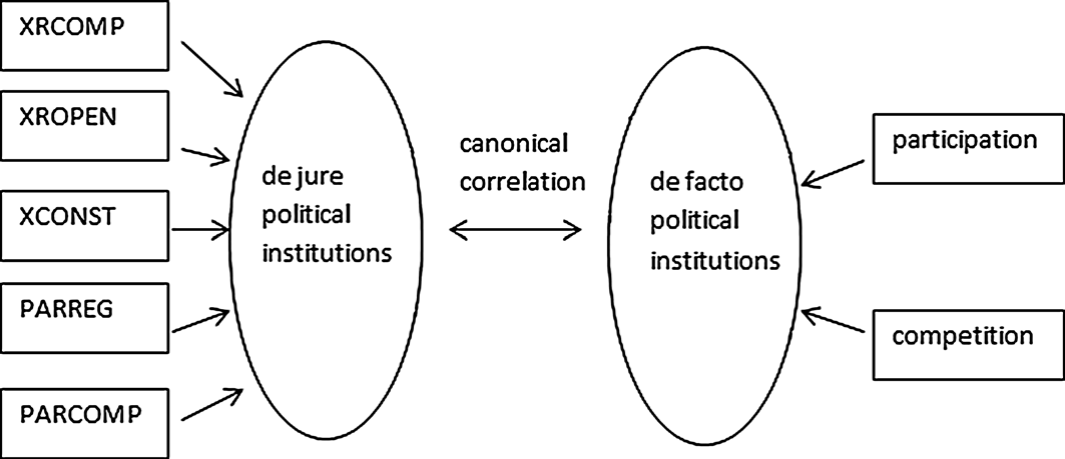
The theoretical outline of the canonical correlation model (the measurement error terms are omitted for simplicity)

The underlying model can be summarized as a block diagram (Fig. 3).

The canonical correlation analysis hence serves as a test for the underlying assumption of this paper, namely that the two sets of variables reflect different dimensions of democracy (de jure and de facto). For periods when we find a high canonical correlation we can argue that the polity2 and the ID convey the same information. If we find little or no relationship, however, the two democracy measures cannot be treated as empirical counterparts of the same latent process.

Unfortunately using canonical correlation has its price too. Since the five components of the polity2 indicator are strongly correlated, we cannot simply create categorical (dummy) variables from all possible outcomes and use them in the canonical correlation analysis due to perfect of near perfect multicollinearity. As an intermediary solution we adopt the arbitrary weighting scheme by Marshal et all (column 4 of Table 3), but we allow each component to have their own weight. While an imperfect solution, this still allows a room for a reweighing of the components and redundant variables will still yield close to zero coefficients. Also, exceptional events, denoted by codes −66, −77 and –88 (foreign interruption, interregnum and transition respectively) in the polity dataset are treated as missing values. We used modern political borders in line with the CLIO-INFRA template; hence the data on historical states that has no obvious equivalent today are omitted as well.

Finally, since we use long-term historical data we also need to cope with the problem of sample selection bias, which is usually neglected in the empirical literature. Namely, the probability that a country is included in the data is not random and is likely to be correlated with the value of the components included in the analysis. Initially we have observations on the developed Western nations such as the USA, the United Kingdom and France, while from the last decades of the nineteenth century we also have data on the periphery to an increasing extent. Also the number of countries increased steadily in the sample period: in 1820 we have only 21 countries for which both the ID and the polityIV has data and the number of observations grows to 151 by 2000. Since countries with more efficient institutions will have a higher chance of being observed (of the 21 countries available in 1820 only 10 are OECD member countries though) than the latecomers the estimated canonical correlation may theoretically be biased upward.

The selection problem has been described by Heckman (1979) as a form of omitted variable bias. We follow his two-step procedure for the canonical correlation analysis. In the first step we estimate the probability if the components of the polity2 and the ID were observed for a particular year conditioned on the subcontinent it is situated on with a probit model.^11^ In the second step we use the two Inverse Mills ratios (on for the ID and another for the polity2 components) estimated in the first step as additional variables in the canonical correlation analysis. The novelty of our approach hence lies in the integration of sample selection correction into the canonical correlation and our historical focus, which allow for the identification of secular changes in the relationship between the two groups of democracy components.

### The Long-Term Movement of the Canonical Correlation Coefficients

**Fig. 4:**
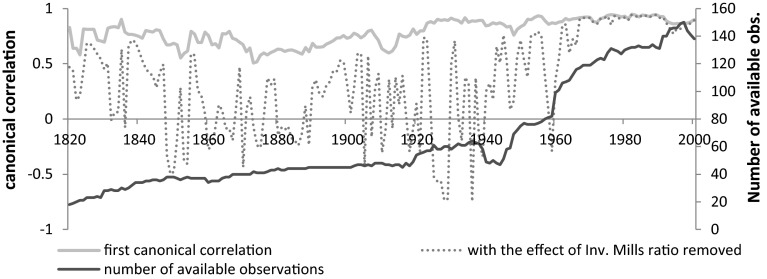
Estimated canonical correlation coefficients per year 1820–2000

The results from the canonical correlation analysis on decadal averages are reported in the “Appendix, in Table 6”. The coefficients suggest that redundancy is indeed a significant issue, since usually only one or two components are found to be significant at at least 10 % level of significance and the rest is usually very close to zero. While our analysis is based on annual estimates, since the results are basically the same, for convenience we report only the decadal estimates in the “Appendix”. The figures are created from the annual estimates for the canonical correlation, however. Figure 4 visualizes both types of canonical correlations, with or without correction for sample selection, and the number of observations.

**Fig. 5:**
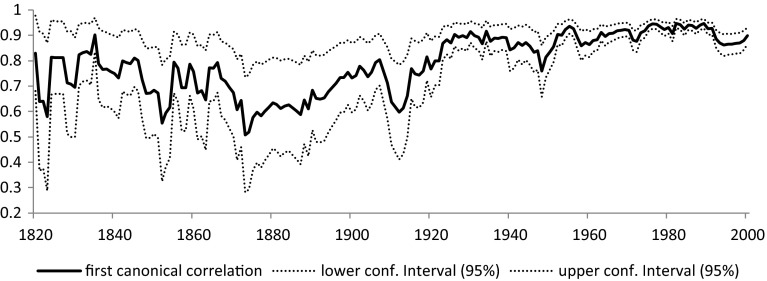
The first canonical correlation coefficient (without correction for sample selection)

Figure 5 reveals a slow increase in the first canonical correlation coefficient^12^ ranging between 0.508 in 1873 and 0.947 in 1982. Even though these estimates are still biased by the selection problem, a trend can already be established. Until about the 1860s the relationship between the two groups of indicators was relatively strong. The relationship began to weaken until 1873 and slowly increased until World War I again. In the 1930s we find a minor setback, followed by World War II, which had a seemingly smaller effect than World War I and the strong correlation gradually restores finally by the 1960s. Even with a possibly upward selection bias present we can conclude that until the twentieth century the relationship between the two set of indicators was medium at most, which confirms the initial assumption that the two indicators capture different aspects of democratization.

Figure 5 tells us a story which is in accordance with standard knowledge about the historical democratization process. De jure political institutions and de facto practices become less connected in periods of fundamental changes or crises such as World War I and II, the Great Depression, when many oppressive regimes came into power and even democracies were forced to take extraordinary measures.

**Fig. 6:**
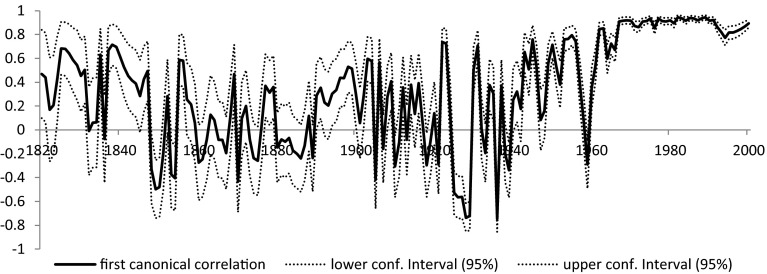
The first canonical correlation coefficient (with correction for sample selection)

Once we correct for sample selection biases, the magnitude of the correlation changes fundamentally (Fig. 6).

**Fig. 7:**
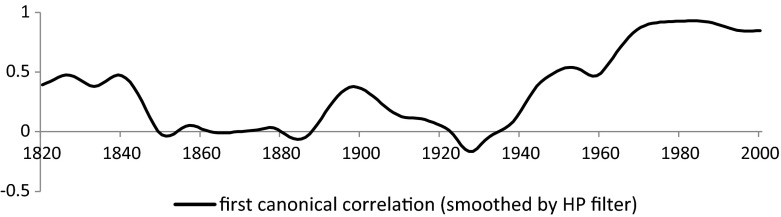
Filtered canonical correlation coefficients (HP filter with *λ* = 100)

In case of the components of the ID, we even have periods when none of the variables yield significant coefficients indicating a complete detachment between de jure and de facto institutions. The corrected canonical correlation coefficients have much larger variation and they become even negative in the 1930s. Yet, one should bear in mind that the probability that a country is included in the sample is also result of an estimation and this introduces additional error. For this reason we apply a Hodrick–Prescot filter (*λ* = 100) on the obtained corrected first canonical correlation coefficients (Fig. 7) to obtain a better interpretable trend. Of course this filtering method is based on certain assumptions: we decided to follow the original paper (Hodrick and Prescott 1997) in choosing the main parameter as 100, which results in smoother results than the alternative (6.25) suggested by Ravn and Uhlig (2002) for annual data.

Figure 7 makes the overall picture easier to see: after an initial, moderate, positive relationship, the corrected canonical correlation coefficients approach zero, meaning that the two groups of components become linearly independent. This is replaced by an upward trend only after World War 2, altogether giving rise to relationship reminiscent of a U-curve. A strong, positive relationship, confirming that the polyarchy dataset by Vanhanen and the Polity IV data by Marshall et al. measure the same underlying process, is only found from the 1970s on.

Table 6 offers an explanation on what is responsible for the pattern observed in Figs. 6 and 7. The inclusion of the inverse Mills ratios capturing the effect of sample selection causes all canonical correlation coefficients to become statistically insignificant from the 1840s until the 1910s and 1920s. Their positive coefficients reflect that basically all observed linear correlation between the components of polity2 and ID in this period is caused by the selection bias. The results are indicative that the degree to which formal rules can translate into outcomes is a time dependent process. Our findings raise doubts regarding the crucial role of changes in de jure political institutions during the early waves of democratization. It is also worth noting that the consistency between the two types of political institutions is a recent phenomenon coinciding with the start of globalization. It is very likely, hence that this high correlation is due to the enforcing power of international markets and organizations, which results in a permanent pressure on indigenous political institutions to adopt foreign-induced, exogenous political institutions.

**Table 6:** Canonical correlation between components of polity2 and ID per decade with and without correction for sample selection bias 1820–1850

	1820s		1830s		1840s		1850s	
*Polity2*								
XRCOMP	0.445 (1.24)	−0.003 (−0.02)	−0.033 (−0.20)	0.016 (0.11)	0.093 (0.38)	0.009 (0–21)	0.027 (0.13)	0.000 (0.01)
XROPEN	−0.069 (−0.13)	−0.075 (−0.24)	−0.247 (−0.72)	−0.120 (−0.39)	−0.408 (−0.80)	−0.068 (−0.73)	−0.172 (−0.37)	0.078 (0.73)
XCONST	0.352^***^ (3.08)	−0.097 (−1.44)	0.493^***^ (7.52)	0.068 (1.19)	0.444^***^ (4.53)	0.003 (0.14)	0.416^***^ (5.00)	0.011 (0.58)
PARREG	0.346 (0.44)	−0.461 (−0.97)	−0.038 (−0.12)	−0.022 (−0.07)	0.158 (0.33)	0.011 (0.13)	−0.330 (−0.76)	−0.107 (−1.06)
PARCOMP	−0.178 (−0.61)	0.135 (0.74)	−0.190 (−1.26)	−0.079 (−0.55)	−0.06 (−0.25)	−0.040 (−0.87)	0.155 (0.68)	0.029 (0.53)
Inv. Mills ratio	–	0.239^***^ (7.11)	–	0.399^***^ (7.53)	–	0.108^***^ (25.0)	–	0.240^***^ (20.6)
*Index of democracy*								
Competition	0.005 (0.21)	0.005 (0.37)	0.081^***^ (7.38)	0.019^*^ (2.01)	0.060^***^ (3.63)	0.004 (0.59)	0.066^***^ (5.66)	0.002 (0.82)
Participation	1.336^***^ (3.72)	−0.369^*^ (−1.81)	−0.117 (−1.27)	−0.064 (−0.80)	0.080 (0.83)	−0.023 (−1.37)	−0.014 −0.29)	0.004 (0.37)
Inv. Mills ratio	–	0.181^***^ (9.38)	–	0.287^***^ (9.88)	–	0.106^***^ (32.8)	–	0.107^***^ (25.5)
Canonical correlation	0.780	0.913	0.840	0.875	0.700	0.985	0.709	0.975
N	24	24	34	34	38	38	38	38

*t*-statistics are reported in parentheses

When inverse Mills ratio is reported, the coefficients are corrected for the selection bias

***,**,* denotes coefficients significant at 10, 5, 1 % level of significance respectively

## Conclusion

The distinction between de jure and de facto political institutions is of primary importance to gain a better insight into the long-waves of democratization. In this paper our point of departure is that the PolityIV and the Index of Democracy measures of democracy should be treated as empirical proxies of de jure and de facto political institutions respectively. We showed that the historical trends of democracy and causal relationship reflected by two measures are so different that the polity2 and the ID aggregates cannot reflect the same underlying latent democracy factor. Also we found that in the two countries where we have a data on de jure political institutions (South Africa and Zimbabwe) de polity2 score is positively and strongly while the ID score is either weakly or even negatively correlated with the de jure political institutions measure.

We used a canonical correlation analysis with correction for sample selection bias to test this hypothesis. The novelty of our approach hence lies in the integration of sample selection correction into the canonical correlation, and in our historical focus. These two allow for the identification of secular changes in the relationship between the two groups of democracy components. We find that after a moderate positive relationship between 1820 and the 1850s the canonical correlation coefficients become insignificant statistically and approach zero. It is not until the 1940s that the relationship starts to recover in global scale and a strong positive statistical relationship is achieved only from the 1970s. Our findings suggest the presence of a U-shaped secular pattern in the relationship between the two democracy measures.

## Appendix: Results from the Canonical Correlation Analysis on Decadal Averages

See Tables 7, 8, 9, and 10.

**Table 7:** Canonical correlation between components of polity2 and ID per decade with and without correction for sample selection bias 1860–1890

	1860s		1870s		1880s		1890s	
*Polity2*								
XRCOMP	−0.037 (−0.19)	0.020 (0.47)	−0.160 (−0.68)	0.024 (0.56)	0.074 (0.32)	0.011 (0.23)	0.279 (1.58)	0.028 (0.53)
XROPEN	0.170 (0.47)	0.042 (0.51)	0.413 (0.90)	−0.004 (−0.05)	0.044 (0.09)	−0.063 (−0.59)	−0.247 (−0.64)	−0.113 (−0.97)
XCONST	0.362^***^ (4.68)	0.007 (0.39)	0.339^***^ (3.63)	0.003 (0.16)	0.306^***^ (3.44)	−0.029 (−1.52)	0.272^***^ (4.47)	−0.026 (−1.38)
PARREG	−0.532 (–1.17)	–0.121 (−1.19)	0.243 (0.52)	−0.078 (−0.91)	–0.122 (−0.23)	0.063 (0.56)	0.458 (1.14)	0.006 (0.05)
PARCOMP	0.296 (1.31)	0.049 (0.98)	0.046 (0.17)	0.041 (0.84)	0.239 (0.85)	−0.077 (−1.32)	−0.143 (−0.69)	−0.050 (–0.80)
Inv. Mills ratio	–	0.253^***^ (24.3)	–	0.260^***^ (25.1)	–	0.197^***^ (20.3)	–	0.195^***^ (20.3)
*Index of democracy*								
Competition	0.053^***^ (5.36)	0.001 (0.41)	0.036^***^ (2.72)	−0.002 (−0.90)	0.046^***^ (3.03)	−0.003 (−1.05)	0.033^***^ (3.14)	−0.001 (−0.28)
Participation	0.045 (1.06)	0.001 (0.07)	0.118^**^ (2.20)	0.001 (0.08)	0.058 (1.06)	−0.003 (−0.26)	0.088^***^ (2.29)	−0.012 (−1.05)
Inv. Mills ratio	–	0.112^***^ (27.7)	–	0.113^***^ (28.0)	–	0.179^***^ (21.4)	–	0.179^***^ (21.0)
Canonical correlation	0.706	0.977	0.630	0.977	0.593	0.964	0.732	0.964
N	42	42	44	44	45	45	45	45

*t*-statistics are reported in parentheses

When inverse Mills ratio is reported, the coefficients are corrected for the selection bias

***,**,* denotes coefficients significant at 10, 5, 1 % level of significance respectively

**Table 8:** Canonical correlation between components of polity2 and ID per decade with and without correction for sample selection bias 1900–1930

	1900s		1910s		1920s		1930s	
*Polity2*								
XRCOMP	0.274^*^ (1.82)	0.020 (0.47)	0.259 (1.64)	0.057 (0.75)	0.046 (0.45)	0.033 (0.75)	−0.044 (−0.57)	−0.016 (−0.41)
XROPEN	−0.746^**^ (−2.37)	0.042 (0.51)	−0.355 (−0.96)	−0.302^*^ (−1.70)	0.414 (1.65)	−0.150 (−1.35)	0.332^*^ (1.88)	−0.017 (−0.19)
XCONST	0.302^***^ (5.26)	0.007 (0.39)	0.197^***^ (3.18)	0.065^**^ (2.13)	0.172^***^ (3.94)	−0.002 (−0.12)	0.312^***^ (9.92)	0.022 (1.37)
PARREG	0.317 (1.08)	−0.121 (−1.19)	0.333 (0.13)	0.045 (0.31)	0.636^***^ (3.05)	−0.114 (−1.19)	0.244 (1.57)	−0.006 (−0.08)
PARCOMP	−0.011 (−0.07)	0.049 (0.98)	0.144 (0.98)	0.030 (0.42)	−0.112 (−1.19)	−0.016 (−0.40)	−0.072 (−0.98)	0.008 (0.22)
Inv. Mills ratio	–	0.253^***^ (24.3)	–	0.265^***^ (14.9)	–	0.266^***^ (26.9)	–	0.291^***^ (36.3)
*Index of democracy*								
Competition	0.045^***^ (5.90)	0.001 (0.41)	0.029^***^ (3.27)	0.005 (1.49)	0.026^***^ (4.88)	−0.000 (−0.03)	0.034^***^ (10.6)	0.003^*^ (1.71)
Participation	0.007 (0.33)	0.001 (0.07)	0.043^**^ (2.52)	0.010 (1.20)	0.025^***^ (2.85)	−0.003 (−0.86)	0.012^**^ (2.58)	0.001 (0–29)
Inv. Mills ratio	–	0.112^***^ (27.7)	–	0.234^***^ (16.6)	–	0.266^***^ (30.9)	–	0.291^***^ (36.3)
Canonical correlation	0.770	0.965	0.741	0.920	0.876	0.972	0.928	0.980
N	50	50	58	58	62	62	65	65

*t*-statistics are reported in parentheses

When inverse Mills ratio is reported, the coefficients are corrected for the selection bias

***,**,* denotes coefficients significant at 10, 5, 1 % level of significance respectively

**Table 9:** Canonical correlation between components of polity2 and ID per decade with and without correction for sample selection bias 1940–1970

	1940s		1950s		1960s		1970s	
*Polity2*								
XRCOMP	0.211^***^ (2.22)	−0.007 −0.84)	0.249^***^ (2.97)	0.042 (1.38)	0.058 (1.09)	−0.016 (−0.34)	0.011 (0.27)	0.058 (1.58)
XROPEN	0.071 (0.30)	0.028 (1.45)	0.337^*^ (1.88)	−0.050 (−0.77)	0.505^***^ (3.70)	0.206^*^ (1.78)	0.154 (1.39)	0.041 (0.43)
XCONST	0.105^***^ (2.57)	0.005 (1.34)	0.062 (1.39)	−0.027^**^ (−1.70)	0.143^***^ (4.62)	0.039 (1.50)	0.102^***^ (3.49)	0.073^***^ (2.89)
PARREG	−0.142 (−0.70)	−0.034^**^ (−2.04)	−0.066 (–0.34)	0.037 (0.52)	0.072 (0.50)	0.005 (0.04)	0.160 (1.08)	0.108 (0.84)
PARCOMP	0.283^***^ (3.06)	0.002 (0.26)	0.138 (1.62)	0.011 (0.35)	0.132^**^ (2.14)	0.057 (1.08)	0.287^***^ (4.68)	0.131^**^ (2.46)
Inv. Mills ratio	–	0.309^***^ (161.3)	–	0.395^***^ (50.3)	–	0.647^***^ (25.5)	–	1.015^***^ (20.9)
*Index of democracy*								
Competition	0.039^***^ (10.4)	−0.000 (−0.73)	0.039^***^ (17.2)	0.001 (0.95)	0.040^***^ (22.2)	0.012^***^ (7.61)	0.040^***^ (27.7)	0.025^***^ (19.7)
Participation	0.006 (1.31)	−0.000 (−0.44)	0.003 (1.04)	−0.003^***^ (−3.44)	0.005^***^ (2.46)	0.001 (0.46)	0.004^***^ (2.73)	−0.002 (−1.14)
Inv. Mills ratio	–	0.312^***^ (173.3)	–	0.460^***^ (52.5)	–	1.466^***^ (26.1)	–	1.409^***^ (20.9)
Canonical correlation	0.886	0.999	0.902	0.986	0.918	0.940	0.958	0.957
N	76	76	84	84	122	122	135	135

*t*-statistics are reported in parentheses

When inverse Mills ratio is reported, the coefficients are corrected for the selection bias

***,**,* denotes coefficients significant at 10, 5, 1 % level of significance respectively

**Table 10:** Canonical correlation between components of polity2 and ID per decade with and without correction for sample selection bias 1980–2000

	1980s		1990s		2000	
*Polity2*						
XRCOMP	0.098^***^ (3.01)	0.079^**^ (2.54)	0.052 (1.06)	−0.058 (−1.27)	0.138 (1.36)	−0.031 (−0.34)
XROPEN	−0.053 (−0.57)	−0.056 (−0.63)	0.029 (0.25)	0.011 (0.11)	0.228 (1.15)	0.027 (0.16)
XCONST	0.110^***^ (4.26)	0.109^***^ (4.43)	0.205^***^ (5.64)	0.210^***^ (6.09)	0.210^***^ (3.10)	0.227^***^ (4.01)
PARREG	0.319^***^ (3.18)	0.173^*^ (1.74)	0.058 (0.50)	−0.305^***^ (−2.80)	−0.074 (−0.40)	−0.146 (−0.93)
PARCOMP	0.167^***^ (3.46)	0.185^***^ (3.99)	0.229^***^ (4.16)	0.260^***^ (4.87)	0.161^*^ (1.90)	0.085 (1.18)
Inv. Mills ratio	–	0.379^***^ (8.49)	–	0.972^***^ (17.1)	–	1.194^***^ (13.9)
*Index of democracy*						
Competition	0.041^***^ (33.4)	0.036^***^ (29.5)	0.041^***^ (20.2)	0.022^**^ (11.2)	0.039^***^ (11.1)	0.018^***^ (5.75)
Participation	0.001 (0.60)	−0.001 (−0.69)	0.004^*^ (1.68)	0.004 (1.62)	0.006 (1.37)	0.001 (0.18)
Inv. Mills ratio	–	0.588^***^ (8.20)	–	2.417^***^ (52.5)	–	2.774^***^ (14.1)
Canonical correlation	0.916	0.962	0.822	0.936	0.821	0.868
N	135	135	158	158	144	139

*t*-statistics are reported in parentheses

When inverse Mills ratio is reported, the coefficients are corrected for the selection bias

***,**,* denotes coefficients significant at 10, 5, 1 % level of significance respectively

## Footnotes

1 The number of countries for which both the PolityIV and the Vanhanen dataset have observation increases over time. In 1820 we have only 21 countries in the sample (Afghanistan, China, Chile, Denmark, France, Haiti, Japan, Morocco, Nepal, the Netherlands, Norway, Oman, Paraguay, Russia, Spain, Sweden, Thailand, Turkey, the UK and the USA. In 2000, on the other hand, we have 151 observations from all continents.

2 The set of de facto and de jure political institutions are hence not disjoint: written rules, when enforced and respected, both classifies as de jure and de facto institutions. Ideally, in a society where all laws and regulations are codified and followed, there is no difference between de jure and de facto institutions, hence it should not matter which one we measure. If there is a detachment between de jure and de facto institutions, then de facto institutions should, however, necessarily be more associated with observed outcomes.

3 The Kuznets curve (Kuznets 1955) establishes an inverse U-shaped relationship between economic development and income inequality.

4 In other words, extractive institutions will not be removed easily which introduces a lag in the process of political transformation.

5 PolityIV project (Marshall et al. 2012) and Vanhanen’s Index of Democracy (Vanhanen 2000, 2003) that are still the most popular datasets on political institutions stretching over the last two centuries. The Polity project is constantly updated and currently has data on 167 countries for 1800–2012, while the Index of Democracy includes 187 countries for 1820–2000.

6 Munck and Verkuilen (2002) already observed a conceptual difference between the two dataset similar to our argument, namely that the polity indicator is more concerned about the regulatory aspects of participation (if the elections are competitive or not), but does not reflect the actual outcomes in term of election results.

7 Our choice was primarily motivated by our goal to use comparable data for the longest possible period. This is the reason why we do not use datasets that are available for shorter periods, such as the Freedom in the World by Freedom House which is available for only after 1972, the Democracy–Dictatorship data (Alvarez et al. 1996; Przeworksy et al. 2000) beginning in 1948 and the Democracy Index by the Economist Intelligence Unit starting from 2006. We also exclude datasets that may have the historical dimension but are binary and hence do not exhibit enough variation for any meaningful multivariate analysis such as Boix et al. (2012) or the Democracy–Dictatorship data which also has this feature.

8 Granger (1969) defines causal link by precedence. If we have reason to assume that the cause happened before the effect, we can use empirical tests regarding the predictive power of time-series on each other to draw conclusion regarding the direction of causality. This is a limited, but empirically feasible definition of causality. Obviously, where expectations play significant role in forming human behaviour, Granger-causality tests are useless. For example, sending Christmas-cards predates Christmas, but no one would believe that those cards actually cause Christmas. In institutional changes, however, this problem is not an issue.

9 See Fedderke et al (2001) and Gwenhamo et al (2012) for the South Africa and Zimbabwe de jure indices respectively.

10 Marshall et al. (2013) warns about the possible shortcomings of their aggregation method in their manual.

11 The regional division follows the Clio-Infra project’s adopted geographical categorization. The regions are: Western and Northern Europe, Eastern Europe (including the USSR and later its successors west of the Ural), Southern Europe, North America, Central America and the Caribbean, South America, Australia and Oceania, East-, West-, South-, Southeast- and Central-Asia, North Africa, and Sub-Saharan Africa.

12 We use and report the first (highest) canonical correlation.
